# Immune Protection Effect of an OmpC-Recombinant T4 Bacteriophage Vaccine Against Infection Caused by Extraintestinal Pathogenic *Escherichia coli* in Mice

**DOI:** 10.3390/vaccines14050383

**Published:** 2026-04-24

**Authors:** Xin Zong, Shiting Ni, Guosheng Chen, Xiaodan Li, Jiaqi Liu, Ze Tong, Zhengnan Yuan, Shiyuan Jiang, Huanchun Chen, Chen Tan, Chenchen Wang

**Affiliations:** 1 National Key Laboratory of Agricultural Microbiology, College of Veterinary Medicine, Huazhong Agricultural University, Wuhan 430070, China; 2 Hubei Hongshan Laboratory, Wuhan 430070, China; 3 Frontiers Science Center for Animal Breeding and Sustainable Production, Wuhan 430070, China; 4 Key Laboratory of Preventive Veterinary Medicine in Hubei Province, Wuhan 430070, China; 5 The Cooperative Innovation Center for Sustainable Pig Production, Wuhan 430070, China

**Keywords:** ExPEC, T4 phage, OmpC epitope, vaccine, immune protection

## Abstract

Background/Objectives: Extraintestinal pathogenic *Escherichia coli* (ExPEC) is a major pathogen that causes septicemia, meningitis, and polyserositis in pigs. The increasing prevalence of antimicrobial resistance and the diverse serotypes of ExPEC highlight the urgent need for broadly protective vaccines. Methods and Results: In this study, an OmpC epitope vaccine based on the T4 phage display system was developed and evaluated. Two B-cell epitopes (OmpC-1 and OmpC-2) were identified by bioinformatic analysis and displayed on recombinant T4 phages. Immunization induced strong antigen-specific IgG responses, with the OmpC-1-T4 group showing significantly higher antibody titers than the OmpC protein group. In the O11 serotype PCN033 challenge model, survival rates reached 100% in the OmpC-1-T4 group, 60% in the OmpC-2-T4 group, and approximately 80% in the OmpC protein group. In the O18 serotype 2103 challenge model, both recombinant phage groups had survival rates of approximately 60%, whereas all the mice in the OmpC protein group died within three days. OmpC-1-T4 immunization also significantly reduced bacterial loads in lung and brain tissues after PCN033 infection and decreased TNF-α and IL-6 expression in lung tissues, accompanied by reduced inflammatory infiltration and tissue damage. Conclusions: Overall, the T4 phage-displayed OmpC epitope vaccine induced strong humoral immunity and provided protection against different ExPEC serotypes. Among the candidates, OmpC-1-T4 showed superior immune protection, bacterial clearance, and inflammation control, supporting its potential as a vaccine candidate against porcine ExPEC infection.

## 1. Introduction

Antimicrobial resistance has become a major challenge to global public health and animal health. According to the World Health Organization, antimicrobial resistance (AMR) directly caused approximately 1.27 million deaths in 2019 and was associated with nearly 4.95 million deaths worldwide [1]. It is projected that, without effective interventions, drug-resistant infections could cause up to 10 million deaths annually by 2050 [2]. This problem is particularly prominent in the livestock industry. *Escherichia coli* (*E. coli*) is an important pathogen responsible for several bacterial diseases in pigs [3]. Among them, extraintestinal pathogenic *E. coli* (ExPEC) can cross the intestinal barrier and infect multiple tissues and organs. It is among the major causative agents of systemic diseases in pigs, including septicemia, polyserositis, arthritis, and meningitis [4]. Epidemiological studies have shown that the proportion of multidrug-resistant (MDR) *E. coli* strains isolated from pigs is continuously increasing [4,5]. Current clinical management of ExPEC infections still relies predominantly on antibiotic therapy. However, resistance rates to ampicillin, tetracycline, sulfonamides, and fluoroquinolones have exceeded 50% in many regions [6]. Moreover, ExPEC strains exhibit high genetic diversity and complex serotype distributions [7]. Several O-antigen serotypes closely associated with extraintestinal infections in pigs have been identified, including O157, O2, O8, O11, O18, O78, O138, and O139 [8]. Marked antigenic diversity complicates surveillance and control and hinders targeted prevention. With declining antimicrobial efficacy and rapid resistance spread, vaccines against porcine ExPEC are urgently needed.

Vaccines can reduce the incidence of infections by inducing specific immune responses, thereby decreasing antibiotic usage and slowing the spread of resistant bacteria [9]. In recent years, several vaccine candidates targeting *E. coli* O antigens, polysaccharides, or surface proteins have been reported [10,11,12]. However, owing to the complex serotype and antigenic diversity of ExPEC, effective vaccines that provide broad protection are lacking [13]. Outer membrane protein C (OmpC), a core member of the outer membrane porin family in gram-negative bacteria, plays a significant role in bacterial physiology, resistance regulation, and host interactions because of its unique structure and function [14,15,16]. It is a key molecule through which porcine *E. coli* adapts to the intestinal environment, initiates infections, and resists external stresses. Like OmpC in other gram-negative bacteria, such as *Salmonella* and *Klebsiella pneumoniae*, OmpC in *E. coli* also has certain immunogenic properties [17,18]. From an antigenic perspective, the N-terminal and C-terminal regions of the OmpC protein contain several conserved B-cell epitopes [19,20]. These epitopes are rich in hydrophilic amino acids and are exposed on the bacterial outer membrane surface, making them easily recognized by host B cells and triggering a humoral immune response and the production of specific IgG and secretory IgA (sIgA) antibodies [21]. Although the development of vaccines based on the *E. coli* OmpC protein for pigs is still in the experimental stage, the OmpC protein of different *E. coli* serotypes shares more than 85% homology, with high sequence specificity [22,23]. This makes it a potential candidate antigen for controlling *E. coli* infections in pigs, which holds significant promise for future applications.

Phage display technology has gained widespread attention because of its ability to display foreign antigens on the surface of phages at high density, its structural stability, and its ease of genetic manipulation [24]. It has gradually been applied in vaccine development. The T4 bacteriophage, a complex and highly stable lytic phage, has a surface that features nonessential outer shell proteins (such as Hoc and Soc) with high plasticity [25]. These proteins can efficiently display foreign antigen molecules through genetic engineering, creating a nanosized antigen delivery platform with good immunogenicity. Previous studies have shown that the T4 phage display system not only significantly improves antigen stability and immune presentation efficiency but also induces strong humoral and cellular immune responses [26,27]. This makes it a promising tool for the development of vaccines against bacteria, viruses, and parasites. Although OmpC is highly conserved in ExPEC and is considered a potential vaccine candidate antigen, previous research has indicated that OmpC-based immune strategies often fail to induce complete immune protection [28]. The cross-protective capacity of OmpC against different ExPEC serotypes remains unclear, and improving antigen immunogenicity remains a key challenge for broad-spectrum vaccine development. Therefore, we constructed a recombinant T4 phage vaccine displaying OmpC and systematically evaluated its immunogenicity and protective efficacy against ExPEC infection, aiming to provide a basis for multiserotype vaccine development.

## 2. Materials and Methods

### 2.1. Bacterial Strains, Phages, and Reagents

The porcine ExPEC O11 serotype strain PCN033 was isolated and preserved at the National Key Laboratory of Agricultural Microbiology Resources Exploration and Utilization, Huazhong Agricultural University. The porcine ExPEC O18 serotype strain 2103 was isolated in 2022 from clinical samples provided by Wuhan Keqian Biology Co., Ltd. (Wuhan, China). The OmpC protein-expressing strain *E. coli* BL21 was preserved in the laboratory. The T4 bacteriophage used for genome editing and its host strain *E. coli* P301 were provided by Pan Tao (Huazhong Agricultural University, Wuhan, China). *E. coli* BL21 and *E. coli* P301 are laboratory-adapted B strains lacking a defined serotype due to defects in O-antigen biosynthesis, resulting in a rough lipopolysaccharide phenotype. The prokaryotic expression vector pET32a(+)-OmpC, pET-RbSoc, the linearized vector pUCSoc-egfp, the donor plasmids pUCSoc-OmpC-1 and pUCSoc-OmpC-2, and the CRISPR-Cas12a editing plasmid were constructed or maintained in our laboratory. Trans Taq DNA polymerase and T4 DNA ligase were purchased from TaKaRa Bio, Inc. (Dalian, China). Plasmid extraction and DNA purification kits were obtained from Omega Bio-Tek (Norcross, GA, USA). Protein quantification kits and TMB substrate were purchased from Beyotime Biotechnology (Shanghai, China). Cesium chloride (CsCl) was obtained from Shanghai Macklin Biochemical Co., Ltd. (Shanghai, China). For Western blot analysis, mouse anti-His monoclonal antibodies and HRP-conjugated goat anti-mouse IgG secondary antibodies were purchased from ABclonal and Abbkine Scientific (Wuhan, China), respectively. The primers used in this study are listed in Table 1.

### 2.2. Prediction of B-Cell Epitopes

Linear B-cell epitopes of the OmpC protein were predicted using the IEDB online analysis tool (http://www.iedb.org/ (accessed on 6 August 2023).) [29]. The full-length amino acid sequence of OmpC was submitted to the server, and epitope prediction was performed using the default parameters. Candidate epitopes with high antigenicity scores were selected for further analysis.

### 2.3. Design of OmpC Multiepitope Genes and Construction of Recombinant Plasmids

The assay was performed as previously described with minor modifications [30]. On the basis of the epitope prediction results, the major antigenic regions of the OmpC protein were selected and linked using a flexible linker (GGGGS) to construct multiepitope sequences. Considering the limited capacity of the donor plasmid for foreign gene insertion, the target sequence was divided into two fragments, designated OmpC-1 and OmpC-2. The coding sequences were optimized according to the codon preference of *E. coli*, chemically synthesized by a commercial company, and cloned and inserted into a T vector for sequence verification. Primers (Soc-F and Soc-R) [31] were designed based on the galSoc gene sequence in the pET-galSoc plasmid to construct the pET-RbSoc plasmid for subsequent experiments. Using the Hoc-Soc-T4 phage donor plasmid pUC-gal-soc as the backbone vector, the linearized vector was obtained by PCR amplification. Recombinant primers containing 15–30 bp homologous arms were designed according to the OmpC-1(i-OmpC-1-F and i-OmpC-1-R) and OmpC-2 (i-OmpC-2-F and i-OmpC-2-R)sequences [28]. The linearized vector (pUCSoc-egfp: linearized vectors were obtained by PCR amplification using inside SOC F and inside SOC R primers [32,33]) and target fragments were amplified using a high-fidelity DNA polymerase. After purification, the PCR products were assembled using a homologous recombination enzyme to generate the recombinant plasmids pUC-Soc-OmpC-1 and pUC-Soc-OmpC-2. The recombinant plasmids were transformed into DH5α competent cells. After heat shock at 42 °C, the transformants were selected on LB agar plates supplemented with ampicillin (Amp^+^). Single colonies were cultured overnight in LB medium, and plasmid DNA was extracted using the FastPure Plasmid Mini Kit (Vazyme). Colony PCR identification was performed using Soc-C FW and ModB primers [34]. Positive clones were further confirmed by Sanger sequencing. The verified recombinant plasmids pUC-gal-Soc-OmpC-1 and pUC-gal-Soc-OmpC-2 were used for subsequent experiments.

### 2.4. Propagation and Purification of the Hoc-Soc-T4 Phage

The assay was performed as previously described with minor modifications [35]. An overnight culture of *E. coli* P301 was inoculated into LB/M9CA medium and incubated at 37 °C until the cell density reached 1.5–2.0 × 10^8^ cells/mL. The bacterial culture was then infected with Hoc-Soc-T4 phage at a multiplicity of infection (MOI) of 0.2–0.4 and incubated at 37 °C for an additional 2–3 h. The culture was subsequently collected. After centrifugation at 30,000× *g* for 30 min, the pellet containing phages was resuspended in Pi-Mg buffer (26 mM Na_2_HPO_4_, 22 mM KH_2_PO_4_, 79 mM NaCl, and 1 mM MgSO_4_) supplemented with chloroform and DNase I. The suspension was incubated at 37 °C for 20 min and then centrifuged at 4300× *g* for 20 min to remove cell debris. The phages in the supernatant were collected by high-speed centrifugation at 30,000× *g* for 30 min. The phage pellet was resuspended in 1 mLmL of Pi-Mg buffer and purified by cesium chloride (CsCl) density gradient centrifugation. The purified phages were dialyzed against dialysis buffer I (10 mM Tris, pH 8.0, 200 mM NaCl, and 5 mM MgCl_2_) for 5 h, followed by overnight dialysis at 4 °C in dialysis buffer II (10 mM Tris, pH 8.0, 50 mM NaCl, and 5 mM MgCl_2_). The endotoxin concentration of the purified Hoc-Soc phage was 1.53 EmL, which is well below the recommended maximum endotoxin concentration of 20 EmL for subunit vaccines.

### 2.5. Sodium Dodecyl Sulfate–Polyacrylamide Gel Electrophoresis (SDS–PAGE) and Western Blot Analysis

The assay was performed as previously described with minor modifications [30]. Recombinant proteins were separated by sodium dodecyl sulfate–polyacrylamide gel electrophoresis (SDS–PAGE) using a 5% stacking gel and a 12% resolving gel (or 10–15% resolving gels depending on the molecular weight of the target protein). Protein samples were denatured by heating in boiling water for 5 min before loading. A volume of 5 μL of protein marker and 5–10 μL of protein sample (not exceeding 15 μL) were loaded onto the gel. Electrophoresis was performed at 100 V for approximately 1.5 h. After electrophoresis, the gels were stained and destained to visualize protein bands. For Western blot analysis, proteins separated by SDS–PAGE were transferred onto PVDF membranes by electroblotting (85 V, 300 mA, 3 h). After transfer, the membranes were blocked with 5% skim milk at room temperature for 1 h. The membranes were then incubated with the corresponding specific antibodies to detect and identify the target proteins.

### 2.6. Animal Immunization and Challenge Experiments

The assay was performed as previously described with minor modifications [28]. All experimental procedures were approved by the Animal Care, Supervision and Control Committee of Huazhong Agricultural University (Approval No. HZAUMO-2024-0098). A total of 45 four-week-old female BALB/c mice were randomly divided into nine groups (n = 5 per group), namely, the OmpC-1-T4 phage vaccine group (two groups), the OmpC-2-T4 phage vaccine group (two groups), the OmpC protein vaccine group (two groups), the adjuvant challenge control group (two groups), and the blank control group (one group). OmpC-1-T4, OmpC-2-T4, and OmpC proteins were mixed with the ISA 15A VG adjuvant and fully emulsified using a vortex mixer. The specific groupings and controls for this study were detailed in Table 2. This vaccination strategy was designed based on previously published studies to ensure adequate immune induction before challenge [28]. Mice in the immunization groups were immunized by multipoint subcutaneous injection on the back, while mice in the adjuvant group received an equal volume of ISA 15A VG adjuvant. A booster immunization was performed on day 14 using the same dose and procedure as the primary immunization. Two weeks after the second immunization, the mice in the OmpC-1-T4 phage vaccine group, OmpC-2-T4 phage vaccine group, OmpC protein vaccine group, and adjuvant challenge control group were challenged by intraperitoneal injection of 2.5 × 10^7^ CFU of PCN033 or 2103 strains per mouse. In this study, tissue samples were collected either at the time of death or at 7 days post-challenge from surviving mice following infection with ExPEC PCN033 or ExPEC 2103. Mice were included in the sampling. And the collected tissues were used for bacterial load determination, inflammatory cytokine analysis, and histopathological examination.

### 2.7. Determination of Antibody Titers

The assay was performed as previously described with minor modifications [36]. OmpC-1-T4, OmpC-2-T4, and OmpC proteins were used as coating antigens on ELISA plates, and antigen-specific antibody levels in mouse serum samples were determined by indirect ELISA. Blood was collected from all 45 mice via the orbital cavity on day 14 after the second immunization, and serum samples were collected by centrifugation. For antigen coating, OmpC-1-T4, OmpC-2-T4, and OmpC proteins were diluted to 2 μg/mL. Afterward, 100 μL of each antigen solution was added to the ELISA plate and incubated overnight at 4 °C. The coating solution was discarded, and the plate was washed three times with 200 μL of PBST per well. Afterward, 200 μL of 5% BSA in PBST was added to each well for blocking at 37 °C for 2 h, followed by washing as described above. For primary antibody incubation, mouse sera were diluted in PBS (pH 7.4) at serial dilutions of 1:200, 1:400, 1:800, 1:1600, 1:3200, 1:6400, 1:12,800, 1:25,600, 1:51,200, 1:102,400, 1:204,800, and 1:409,600. A volume of 100 μL of diluted serum was added to each well and incubated at 37 °C for 1 h, followed by washing. For secondary antibody incubation, HRP-conjugated goat anti-mouse IgG was diluted 1:8000, and 100 μL was added to each well and incubated at 37 °C for 1 h, followed by washing. Finally, 100 μL of TMB substrate solution was added to each well and incubated at room temperature in the dark for 15 min. The reaction was stopped by adding 100 μL of stop solution, and the absorbance was measured at 450 nm using a microplate reader.

### 2.8. Determination of Bacterial Loads in Tissues

After bacterial challenge, the mice were euthanized under aseptic conditions, and the brain and lung tissues were collected for bacterial load determination. The tissues were weighed and placed into sterile homogenization tubes containing an appropriate volume of sterile PBS and mechanically homogenized. The resulting tissue homogenates were serially diluted 10-fold to a concentration of 10^−8^. Appropriate dilutions were plated onto LA agar plates and incubated overnight at 37 °C. After incubation, the number of colony-forming units (CFUs) on the plates was determined, and the bacterial loads were calculated as CFU per gram of tissue (CFU/g) on the basis of the dilution factor and tissue weight.

### 2.9. Measurement of Inflammatory Cytokine Expression

The expression levels of the inflammatory cytokines TNF-α and IL-6 were determined by quantitative real-time PCR (qRT–PCR). Total RNA was extracted from the lung tissue samples and reverse transcribed to generate cDNA. Specific primers for TNF-α, IL-6, and the housekeeping gene GAPDH were designed on the basis of mouse gene sequences. The qRT–PCRs were performed using a SYBR Green I detection system with a total reaction volume of 10 μL. Each sample was analyzed in three technical replicates. Amplification was performed using a real-time PCR instrument, and fluorescence signals were recorded during the reaction. The relative expression levels of the target genes were calculated using the 2^−ΔΔCt^ method.

### 2.10. Histopathological Analysis

Mouse lung tissues were collected for histological examination. Fresh lung tissues were fixed in 10% neutral buffered formalin for at least 24 h. The tissues were then dehydrated, embedded in paraffin, and sectioned into 4 μm slices. The sections were stained with hematoxylin and eosin (H&E). Histopathological changes, including changes in alveolar structure, alveolar septal thickness, and inflammatory cell infiltration, were examined under a light microscope.

### 2.11. Statistical Analysis

All the experimental data were analyzed using the software GraphPad Prism 9.0. The results are presented as the mean ± standard deviation (mean ± SD). Each in vitro experiment was performed at least three times independently, and the sample size for the animal experiments is indicated in the corresponding figure legends. Differences between two groups were analyzed using Student’s *t* test. Comparisons among three or more groups were performed using one-way analysis of variance (one-way ANOVA), followed by appropriate post hoc multiple comparison tests when overall differences were significant. For bacterial colony count data, such as tissue bacterial loads, values were log10-transformed before statistical analysis. Survival curves were generated using the Kaplan–Meier method and compared using the log-rank test. Relative gene expression in the qRT–PCR assays was calculated using the 2^−ΔΔCt^ method and normalized to that of the housekeeping gene. All the statistical tests were two-tailed, and differences were considered to be statistically significant when *p* < 0.05.

## 3. Results

### 3.1. Screening of OmpC Epitopes and Successful Display on the T4 Phage Surface

**Figure 1:**
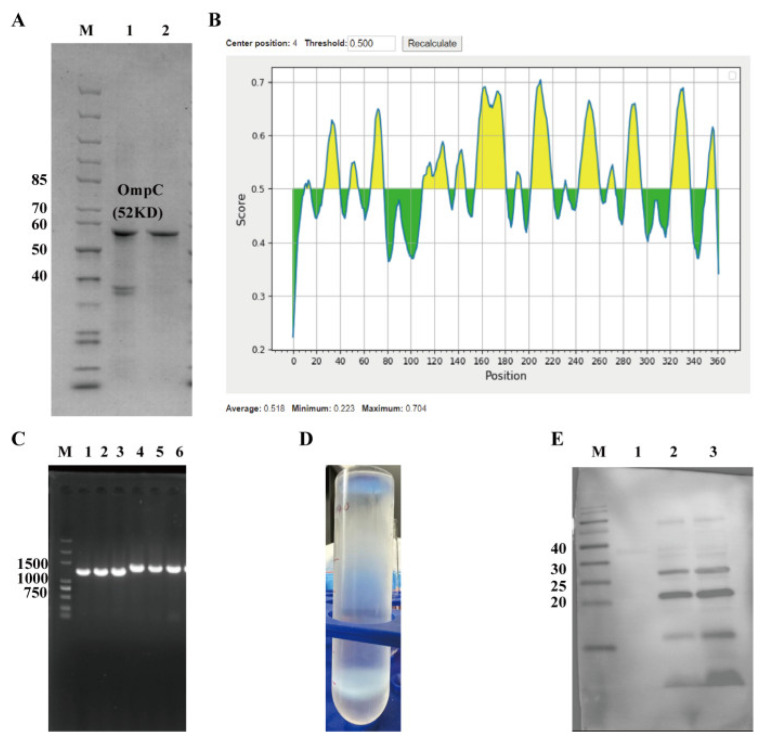
Screening of OmpC Antigen Epitopes and Construction and Validation of Recombinant T4 Phages. (**A**) SDS–PAGE analysis of OmpC protein expression and refolding purification. M: protein molecular weight marker; 1: refolded precipitate; 2: refolded supernatant. (**B**) Prediction of B-cell antigenic epitopes of the OmpC protein. The x-axis represents the amino acid sequence position, and the y-axis represents the antigenicity score. A threshold of 0.5 was set, with regions above the threshold predicted as potential antigenic epitopes. (**C**) PCR validation of the integration of the target gene during recombinant phage construction. M: DNA molecular weight marker; 1–3: OmpC-1; 4–6: OmpC-2. (**D**) Amplification and CsCl density gradient centrifugation purification results of the recombinant T4 phage, showing distinct phage bands. (**E**) Western blot detection of OmpC antigenic epitopes displayed on the recombinant T4 phage. M: protein molecular weight marker; 1: control; 2: OmpC-1 epitope; 3: OmpC-2 epitope.

To obtain an OmpC antigen suitable for T4 phage display and identify immunogenic epitopes, OmpC expression, purification, and epitope prediction were performed. The protein was successfully expressed in E. coli and purified as a refolded inclusion body using glutathione affinity chromatography. SDS–PAGE showed a clear band at ~52 kDa, consistent with the expected molecular weight (Figure 1A). Lane 1 represents the refolded precipitate, and lane 2 the refolded supernatant, where the presence of the target band indicates successful partial refolding into a soluble form. Bioinformatic analysis identified multiple antigenic regions with scores > 0.5 (Figure 1B), and two candidate B-cell epitopes, OmpC-1 and OmpC-2, were selected. These sequences were successfully inserted into the T4 phage Soc-Hoc site (Figure 1B), as confirmed by PCR (Figure 1C) and sequencing (Figure S1). Following plaque screening, amplification, and CsCl gradient purification, distinct phage bands were observed (Figure 1D). Western blot further confirmed specific immune-reactive bands, demonstrating successful surface display and antibody recognition of OmpC-1 and OmpC-2 (Figure 1E). These results confirm the successful construction of recombinant T4 phages displaying OmpC epitopes, providing a basis for subsequent immunogenicity and protection studies.

### 3.2. Recombinant T4 Phage Displaying OmpC Epitopes Induces Significant IgG Antibody Responses

**Figure 2:**
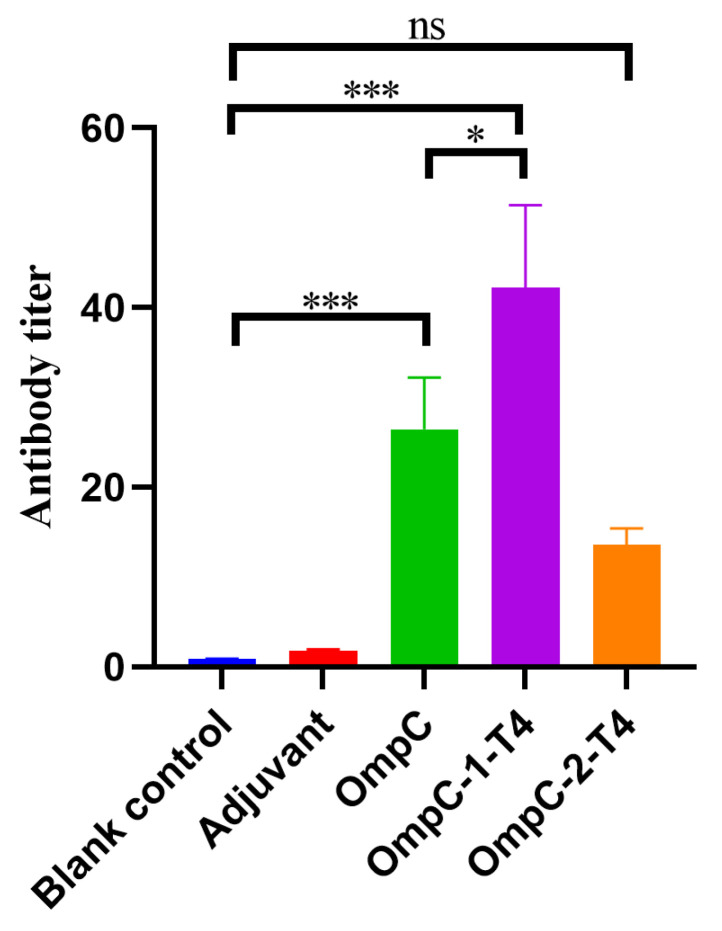
IgG Antibody Titers in Mice Induced by Two OmpC Epitope-Displaying Recombinant T4 Phages. Serum samples were collected from the mice on day 14 after the second immunization, and specific IgG antibody titers were measured by ELISA. Blank control: blank control group; Adjuvant: adjuvant control group; OmpC: OmpC protein immunization group; OmpC-1-T4 and OmpC-2-T4: recombinant T4 phage immunization groups displaying the OmpC-1 and OmpC-2 epitopes, respectively. The data are presented as the mean ± SD. Statistical analysis was performed using one-way analysis of variance (one-way ANOVA). * *p* < 0.05; *** *p* < 0.001. ns indicates no significant difference.

To evaluate the immune responses induced by the two OmpC epitope-displaying recombinant T4 phages, serum samples were collected from mice on day 14 after the second immunization, and specific IgG antibody titers were measured by ELISA. As shown in Figure 2, the blank control group and the adjuvant control group produced only very low levels of antibody responses. Compared with the control groups, the OmpC protein immunization group exhibited a clear antibody response (*** *p* < 0.001) (Figure 2). Among the two recombinant phage immunization groups, the OmpC-1-T4 group generated the highest antibody titers. The titers were significantly higher than those in the OmpC protein group (* *p* < 0.05) and the control groups (*** *p* < 0.001) (Figure 2). In contrast, antibody production was induced in the OmpC-2-T4 group, but the antibody titers were lower than those in the OmpC-1-T4 group (Figure 2). Statistical analysis revealed a significant difference between the OmpC-1-T4 and OmpC-2-T4 groups, whereas no significant difference (ns) was detected between the control groups (Figure 2). These results indicated that both OmpC epitope-displaying recombinant T4 phages could induce specific humoral immune responses, with OmpC-1-T4 showing stronger immunogenic effects.

### 3.3. Recombinant T4 Phage Vaccines Provide Immune Protection Against Different Serotypes of ExPEC Infection

**Figure 3:**
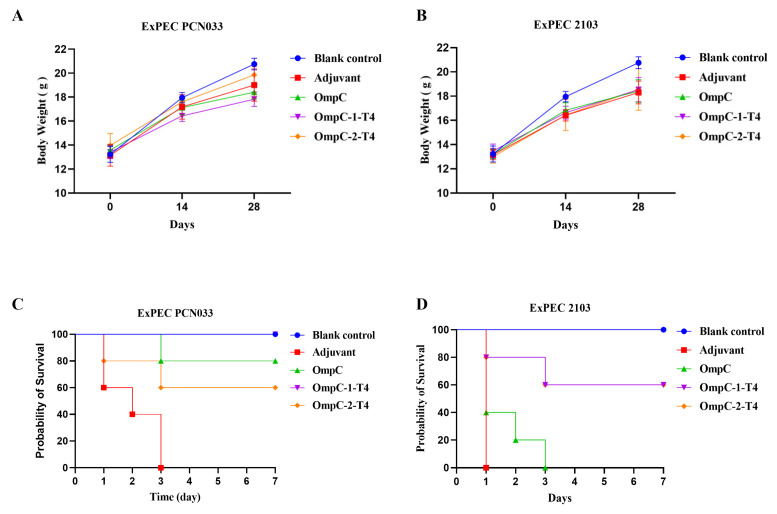
Immune Protective Effects of Recombinant T4 Phage Vaccines Against Different Serotypes of Porcine ExPEC Infection. (**A**,**B**) Changes in body weight of the mice during the immunization period. Body weights were recorded on days 0, 14, and 28 after immunization to evaluate the effect of immunization on mouse growth. (**A**) Body weight changes in mice corresponding to the ExPEC PCN033 challenge experiment. (**B**) Body weight changes in mice corresponding to the ExPEC 2103 challenge experiment. (**C**,**D**) Survival curves of mice after bacterial challenge. Mice were challenged on day 28 after immunization with the porcine ExPEC O11 serotype strain PCN033 or the O18 serotype strain 2103. Mouse survival was monitored for 7 days. (**C**) ExPEC PCN033 challenge model. (**D**) ExPEC 2103 challenge model.

To evaluate the protective efficacy of the two OmpC epitope-displaying recombinant T4 phages against porcine ExPEC, challenge experiments were performed on day 28 after immunization using the O11 serotype strain PCN033 and the O18 serotype strain 2103. Body weight changes were first monitored during the immunization period to assess vaccine safety and its effect on normal growth (Figure 3A,B). Throughout the immunization period, the body weights of the mice in all the groups gradually increased over time. No abnormal changes or significant differences were observed among the groups, indicating that the different immunization treatments did not adversely affect normal growth (Figure 3A,B; Table 3). The mice were challenged on day 28 after immunization, and survival was recorded (Table 4). In the PCN033 challenge model, all the mice in the adjuvant control group died within three days (Figure 3C). The OmpC protein-immunized group showed partial protection, with a survival rate of approximately 80% (Figure 3C). In comparison, OmpC-1-T4 immunization had the strongest protective effect, with a survival rate of 100% (Figure 3C). The OmpC-2-T4 group showed moderate protection, with a final survival rate of approximately 60% (Figure 3C). In the 2103 challenge group, the protection provided by OmpC protein was weak, and all the mice died within three days (Figure 3D). In contrast, both recombinant phage immunization groups displayed clear protective effects (Figure 3D). The final survival rates of the OmpC-1-T4 and OmpC-2-T4 groups were both 60% (Figure 3D). Overall, recombinant T4 phages displaying OmpC epitopes conferred protection against O11 and O18 ExPEC serotypes, with OmpC-1-T4 showing stronger efficacy in the PCN033 challenge model.

### 3.4. Recombinant T4 Phages Displaying OmpC Epitopes Reduce Tissue Bacterial Loads After ExPEC Infection

**Figure 4:**
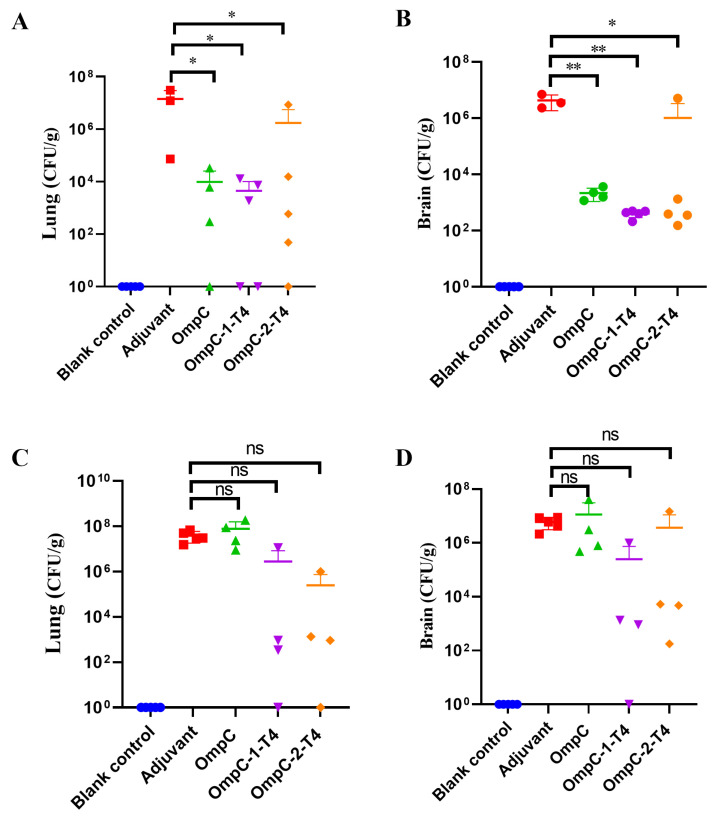
Bacterial Loads in the Lung and Brain Tissues of Mice after Immunization. Mice were challenged on day 28 after immunization with the porcine extraintestinal pathogenic *E. coli* (ExPEC) O11 serotype strain PCN033 or O18 serotype strain 2103. Bacterial loads in lung and brain tissues were then measured (lg CFU/g). (**A**,**B**) Bacterial loads in the lung tissue (**A**) and brain tissue (**B**) of mice in the PCN033 challenge model. (**C**–**D**) Bacterial loads in the lung tissue (**C**) and brain tissue (**D**) of mice in the 2103 challenge model. The data are presented as the mean ± SD. Statistical analysis was performed using one-way analysis of variance (one-way ANOVA). * *p* < 0.05; ** *p* < 0.01. ns indicates no significant difference.

To further evaluate the ability of the immunized mice to control ExPEC infection, bacterial loads in the lung and brain tissues were measured after challenge (Table 5). In the PCN033 challenge model, compared with those in the adjuvant control group, the bacterial loads in the lungs of mice in all immunized groups were reduced (Figure 3A). Among these groups, the OmpC-1-T4 immunization group presented a significant reduction in the bacterial load in lung tissue (* *p* < 0.05) (Figure 4A). The OmpC protein group and the OmpC-2-T4 group also tended to decrease (Figure 4A). Similarly, compared with those in the control group, the bacterial loads in the OmpC protein group and the two recombinant phage groups were markedly lower (Figure 4B). The reduction was most obvious in the OmpC-1-T4 group and reached statistical significance (* *p* < 0.05 or ** *p* < 0.01), suggesting that this vaccine could effectively inhibit bacterial dissemination in the host (Figure 4B). In the 2103 challenge model, the overall trend in tissue bacterial loads among the different immunization groups was similar to that observed in the PCN033 model, but the differences did not reach statistical significance (Figure 4C,D). In the lung tissue, the bacterial loads in the OmpC-1-T4 and OmpC-2-T4 groups were lower than those in the adjuvant control group, but the differences were not significant (ns) (Figure 4C). In the brain tissue, the bacterial loads of the recombinant T4 phage groups also tended to decrease. The overall level in the OmpC-1-T4 group was lower, but statistical analysis revealed no significant difference among the groups (Figure 4D). Overall, immunization with recombinant T4 phages displaying OmpC epitopes reduced the colonization of lung and brain tissues by ExPEC more effectively than immunization with the OmpC protein alone did.

### 3.5. Recombinant T4 Phages Displaying OmpC Epitopes Reduce Pulmonary Inflammation After ExPEC Infection

**Figure 5:**
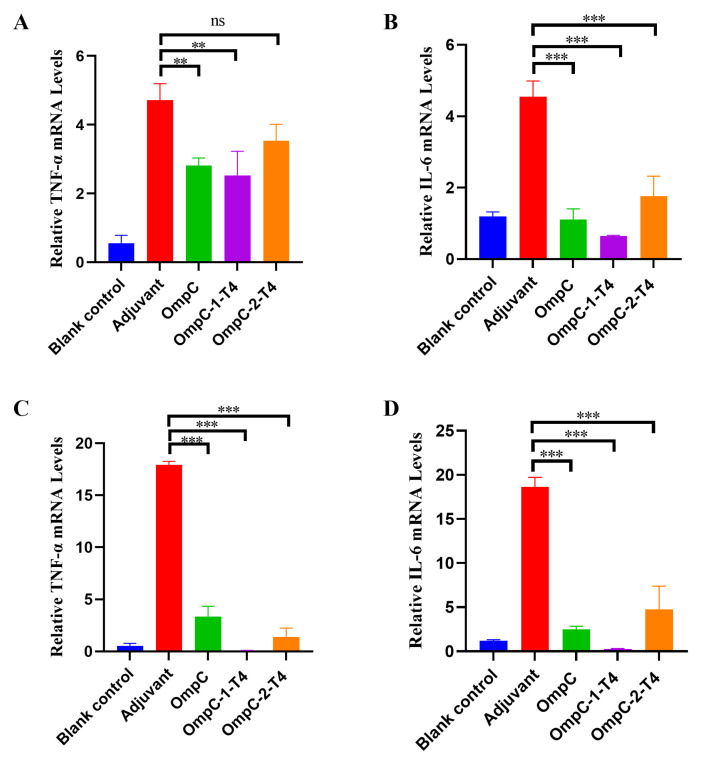
Expression Levels of Inflammatory Cytokines in Mouse Lung Tissues after Immunization. Mice were challenged on day 28 after immunization with the porcine ExPEC O11 serotype strain PCN033 or O18 serotype strain 2103. The mRNA expression levels of the inflammatory cytokines TNF-α and IL-6 in lung tissues were measured by qRT–PCR. (**A**,**B**) Relative mRNA expression levels of TNF-α (**A**) and IL-6 (**B**) in the PCN033 challenge model. (**C**,**D**) Relative mRNA expression levels of TNF-α (**C**) and IL-6 (**D**) in the 2103 challenge model. The data are presented as the mean ± SD. Statistical analysis was performed using one-way analysis of variance (one-way ANOVA). ** *p* < 0.01; *** *p* < 0.001. ns indicates no significant difference.

To further evaluate the inflammatory response after ExPEC infection, the mRNA expression levels of the inflammatory cytokines TNF-α and IL-6 in lung tissues were measured after bacterial challenge. In the PCN033 challenge model, the expression levels of TNF-α and IL-6 in the lung tissues of the adjuvant control group were significantly greater than those in the lung tissues of the blank control group, indicating that bacterial infection induced a strong inflammatory response (Figure 5A,B). Among them, the OmpC-1-T4 immunization group had the lowest expression levels of TNF-α and IL-6, which were significantly lower than those in the adjuvant control group (*p* < 0.01 or *** *p* < 0.001) (Figure 5A,B). The OmpC protein group and the OmpC-2-T4 group also tended to decrease (Figure 5A,B). In the 2103 challenge model, the changes in cytokine expression were similar to those observed in the PCN033 model. The expression of TNF-α and IL-6 in lung tissue significantly increased in the adjuvant control group, whereas inflammation clearly decreased in the immunized groups (Figure 5C,D). Among these groups, the OmpC-1-T4 group showed the strongest inhibitory effect on inflammatory cytokines. The expression levels of TNF-α and IL-6 were significantly lower than those in the adjuvant control group and the OmpC protein group (*** *p* < 0.001) (Figure 5C,D). In comparison, the cytokine expression in the OmpC-2-T4 group was reduced to some extent, but the overall inhibitory effect was weaker than that in the OmpC-1-T4 group (Figure 5C,D). These results indicated that recombinant T4 phages displaying OmpC epitopes reduced pulmonary inflammation after ExPEC infection, with OmpC-1-T4 more effectively suppressing inflammatory cytokine expression.

### 3.6. Recombinant T4 Phages Displaying OmpC Epitopes Alleviate Lung Tissue Damage Induced by ExPEC Infection

**Figure 6:**
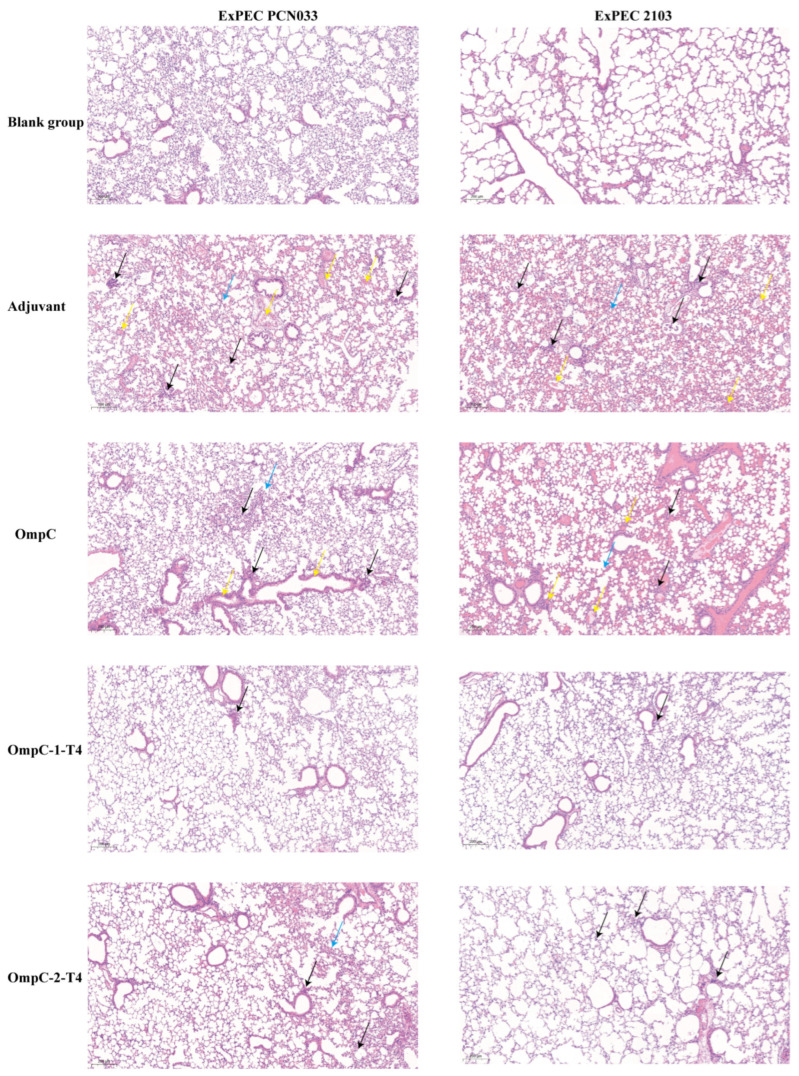
Histopathological observation of mouse lung tissues after ExPEC challenge by H&E staining. Mice were challenged on day 28 after immunization with the porcine extraintestinal pathogenic *E. coli* (ExPEC) O11 serotype strain PCN033 or O18 serotype strain 2103. Lung tissues were collected for hematoxylin and eosin (H&E) staining and histopathological examination. The left panel shows the PCN033 challenge model, and the right panel shows the 2103 challenge model. Scale bar: 200 µm. Yellow arrows indicate tissue congestion, black arrows indicate inflammatory cell infiltration, and blue arrows indicate septal swelling.

To further evaluate lung tissue injury after ExPEC infection, lung tissues from challenged mice were examined by hematoxylin and eosin (H&E) staining and histopathological analysis. In both the PCN033 and 2103 challenge models, lung tissues from the blank control group showed intact structures, clear alveolar architecture, and no obvious inflammatory cell infiltration (Figure 6). In contrast, mice in the adjuvant control group exhibited marked pathological damage to their lung tissue (Figure 6). This damage was characterized by disordered alveolar structures, obvious thickening of the alveolar septa, and extensive infiltration of inflammatory cells, indicating that bacterial infection induced a strong pulmonary inflammatory response (Figure 6). In the OmpC protein immunization group, lung tissue damage was reduced compared with that in the adjuvant control group, although moderate inflammatory cell infiltration and structural changes in the alveoli were still observed (Figure 6). In comparison, the two OmpC epitope-displaying recombinant T4 phage immunization groups showed stronger tissue protective effects (Figure 6). In the OmpC-1-T4 group, the lung tissue structure was largely preserved, with only slight thickening of the alveolar septa and markedly reduced inflammatory cell infiltration (Figure 6). The OmpC-2-T4 group also showed partial alleviation of tissue damage. In the 2103 challenge model, the overall pathological changes were similar to those observed in the PCN033 model. Among all the groups, the lung tissue structure of the OmpC-1-T4 immunization group was the closest to that of the normal group (Figure 6). These results indicated that recombinant T4 phages displaying OmpC epitopes alleviated ExPEC-induced lung inflammation, with OmpC-1-T4 showing stronger protection.

## 4. Discussion

### 4.1. OmpC as a Vaccine Antigen

Outer membrane proteins are highly exposed on the surface of gram-negative bacteria and participate in material transport, host adhesion, and immune recognition [14,15,16]. Therefore, they have long been considered important candidate antigens for the development of broad-spectrum vaccines. The sequence conservation of OmpC, one of the major porins of *E. coli*, is relatively high among different strains, and it is regarded as a potential antigen with cross-protective value [28]. However, previous studies have shown that vaccines based on outer membrane proteins can induce antibody responses, but the protection rate against some strains is only 50–75% [28,37]. These findings suggest that the immune protection provided by a single antigen is still limited. In addition, structural studies show that although OmpC is generally conserved, variations in its surface loops may affect antibody recognition efficiency [38]. Therefore, enhancing OmpC immunogenicity and optimizing antigen presentation are critical for improving vaccine efficacy.

### 4.2. The Advantage of T4 Phage Display System

In this study, two potential B-cell epitopes were identified by bioinformatic analysis and displayed using the T4 phage system. The Hoc and Soc proteins on the surface of T4 phage can display foreign antigens at high density and form virus-like particle structures [39]. This arrangement can significantly increase antigen immunogenicity. Previous studies have shown that, compared with conventional protein immunization, T4 phage-displayed antigens can induce markedly higher antibody titers, sometimes exceeding a tenfold increase [40,41]. In this study, OmpC epitopes were successfully displayed on the T4 phage surface. The recombinant phages induced strong IgG responses, with OmpC-1-T4 eliciting the highest titers, indicating that the T4 display system enhances OmpC immunogenicity and highlights its potential for bacterial vaccine development.

### 4.3. Protective Efficacy Against O11 and O18 ExPEC Serotypes

In the evaluation of immune protection, two porcine ExPEC strains, O11 serotype PCN033 and O18 serotype 2103, were used for challenge experiments to assess the protective ability of the vaccine against different serotypes. Because of the high diversity of ExPEC serotypes and virulence factors, the development of vaccines with broad protective capacity remains challenging [42]. The results of this study revealed that the protection rate of the OmpC-1-T4-immunized group was 100% in the PCN033 challenge model, whereas that of the OmpC-2-T4-immunized group was 60%. In the 2103 challenge model, both recombinant phage vaccines provided approximately 60% protection. Compared with previous studies, this level of protection has certain advantages [28]. In addition, tissue bacterial loads were assessed. The results showed that bacterial burdens in the lung and brain were significantly reduced in the OmpC-1-T4-immunized group after PCN033 infection, with a similar decreasing trend observed in the 2103 model. These findings indicate that the OmpC epitope-displaying phage vaccine not only improves survival but also limits bacterial dissemination. Notably, stronger protection was observed in the PCN033 model, whereas efficacy declined in the 2103 model, possibly due to antigenic variation or differences in virulence factor expression among serotypes. Overall, the vaccine demonstrates potential for O11and O18 serotype ExPEC protection.

### 4.4. Modulation of Inflammatory Responses and Tissue Protection

Inflammatory responses are among the major causes of tissue damage during ExPEC infection [43]. After bacterial infection, the host immune system rapidly releases inflammatory cytokines such as TNF-α and IL-6 to promote immune cell recruitment and pathogen clearance. However, excessive inflammation can lead to severe tissue damage and organ dysfunction [44]. In this study, TNF-α and IL-6 levels in lung tissues were significantly elevated in the adjuvant control group after bacterial challenge, accompanied by marked tissue damage and inflammatory cell infiltration. In contrast, these cytokine levels were markedly reduced in the OmpC epitope displaying T4 phage groups, with significantly alleviated lung injury. These findings indicate that vaccination effectively attenuates infection-induced inflammatory responses. Notably, the OmpC-1-T4 group showed stronger anti-inflammatory and histopathological improvements, consistent with its higher antibody titers and enhanced protective immunity. Previous studies have shown that vaccine-induced specific antibodies can reduce bacterial burden by promoting complement activation and enhancing phagocytic clearance. This process ultimately helps to limit inflammation-associated tissue damage [45].

### 4.5. Limitations and Future Perspectives

However, this study has several limitations. First, because the porcine infection model for ExPEC is not yet fully established in our laboratory. Therefore, the protective efficacy of the vaccine was evaluated only in a mouse model. Thus, further studies in natural hosts such as pigs are needed to confirm its protective effect. Second, this study focused mainly on humoral immune responses, while cellular immunity plays an important role in antibacterial defense. Future work should evaluate T-cell responses and cytokine profiles. In addition, only two OmpC antigenic epitopes were screened and validated in this study, and other epitopes with higher immunogenic potential may exist. Future studies using multiepitope vaccines or combining OmpC with other conserved antigens may further improve broad-spectrum protection. Overall, for the first time, the construction and validation of an OmpC epitope vaccine based on the T4 phage display system. The vaccine induces effective immune responses and provides partial protection against multiple ExPEC serotypes, offering a promising strategy for the development of vaccines against porcine ExPEC infection.

## 5. Conclusions

In short, the OmpC epitope vaccine constructed using the T4 phage display system induced a strong humoral immune response and provided a certain level of protection against infections caused by O11and O18 serotype ExPEC. Among the tested vaccines, OmpC-1-T4 showed better immune protection, bacterial clearance, and suppression of inflammatory responses. These findings provide experimental support for the development of a candidate vaccine against porcine ExPEC infection.

## Figures and Tables

**Table 1:** The primers used in this study.

Primers	Primer Sequence (5′-3′)
Soc-F	CTAGCTAGC GGTGGTTATGTAAACATCAAAAC
Soc-R	CCGCTCGAG ACCACTTACTGGTGTAG
SOC-C FW	taatttacttattcagaaaagaagatgtataaataatca
ModB	gaaagtataatatcgctgatatggt
i-OmpC-1-F	gtaactggtCTCGAGGGCGGTGGCGGTAGCAACAAAGACGGCAAC
i-OmpC-1-R	gagtccttgagttattaACCGTCACCGTTCTG
i-OmpC-2-F	gtaactggtCTCGAGGGCGGTGGCGGTAGCAATTATGAAGGCTTT
i-OmpC-2-R	gagtccttgagttattaAGTGTTGATGCCAGC
inside SOC F	taataactcaaggactccttcgggagtccttttttc
inside SOC R	CTCGAGaccagttactttccacaaatcttcatttgcagc
Mouse-GAPDH-F	CACGGCAAATTCAACGGCACAGTC
Mouse-GAPDH-R	ACCCGTTTGGCTCCACCCTTCA
Mouse-IL-6-F	TCCAGTTGCCTTCTTGGGAC
Mouse-IL-6-R	GTACTCCAGAAGACCAGAGG
Mouse-TNF-α-F	AGCCCACGTCGTAGCAAACCAC
Mouse-TNF-α-R	ACACCCATTCCCTTCACAGAGC

**Table 2:** Immune grouping and immunological dose.

Group	Number	Antigen Dose	Bacterial Attack
OmpC-1-T4	5	50 μg (protein:adjuvant 85:15 volume ratio)	ExPEC PCN033
	5	50 μg (protein:adjuvant 85:15 volume ratio)	ExPEC 2103
OmpC-2-T4	5	50 μg (protein:adjuvant 85:15 volume ratio)	ExPEC PCN033
	5	50 μg (protein:adjuvant 85:15 volume ratio)	ExPEC 2103
OmpC	5	50 μg (protein:adjuvant 85:15 volume ratio)	ExPEC PCN033
	5	50 μg (protein:adjuvant 85:15 volume ratio)	ExPEC 2103
Adjuvant	5	Equal volume (PBS: adjuvant 85:15 volume ratio)	ExPEC PCN033
	5	Equal volume (PBS: adjuvant 85:15 volume ratio)	ExPEC 2103
Blank control	5	-	-

**Table 3:** Changes in body weight of the mice during the immunization period.

Group	ExPEC PCN033			ExPEC 2103		
	Body Weight (g)					
	0 Day	14 Day	28 Day	0 Day	14 Day	28 Day
Blank control	13.23 ± 0.66	17.95 ± 0.43	20.76 ± 0.50	13.23 ± 0.66	17.95 ± 0.43	20.76 ± 0.50
Adjuvant	13.14 ± 0.86	17.1 ± 0.98	19.00 ± 1.37	13.16 ± 0.41	16.41 ± 0.47	18.30 ± 0.89
OmpC	13.58 ± 0.45	17.13 ± 0.61	18.42 ± 0.60	13.23 ± 0.40	16.83 ± 0.64	18.42 ± 0.95
OmpC-1-T4	13.41 ± 0.54	16.44 ± 0.49	17.83 ± 0.57	13.48 ± 0.58	16.64 ± 0.48	18.53 ± 1.02
OmpC-2-T4	13.97 ± 0.99	17.59 ± 0.35	19.85 ± 0.68	13.00 ± 0.52	16.44 ± 1.20	18.56 ± 1.62

**Table 4:** Survival curves of mice after bacterial challenge.

Bacterial Strain	Blank Control	Adjuvant	OmpC	OmpC-1-T4	OmpC-2-T4
	Survival Rate (%) of Mice Within 7 Days				
ExPEC PCN033	100	0	80	100	60
ExPEC 2103	100	0	0	60	60

**Table 5:** Bacterial loads in the lung and brain tissues of mice after immunization.

Group	ExPEC PCN033		ExPEC 2103	
	Tissue Bacterial Load (lgCFU/g)			
	Lung	Brain	Lung	Brain
Blank control	0	0	0	0
Adjuvant	6.48 ± 1.42	6.59 ± 0.24	7.54 ± 0.25	6.59 ± 0.24
OmpC	2.70 ± 2.03	3.30 ± 0.21	7.89 ± 0.27	3.30 ± 0.21
OmpC-1-T4	2.25 ± 2.05	2.59 ± 0.15	3.13 ± 2.98	2.59 ± 0.15
OmpC-2-T4	3.11 ± 2.60	3.43 ± 1.91	3.522 ± 2.27	3.43 ± 1.91

## Data Availability

The data presented in this study are available on request from the corresponding author.

## Supplementary Materials

The following supporting information can be downloaded at: https://www.mdpi.com/article/10.3390/vaccines14050383/s1, Figure S1: OmpC antigen epitope sequencing results.

## References

[1] Hussain A., Najeeb A., Ali S.A. (2025). Antimicrobial Resistance: A Modern Plague. AI-Driven Breakthroughs in Antimicrobial Resistance.

[2] Salam M.A., Al-Amin M.Y., Salam M.T., Pawar J.S., Akhter N., Rabaan A.A., Alqumber M.A. (2023). Antimicrobial resistance: A growing serious threat for global public health. Healthcare.

[3] Abubakar R.H., Madoroba E., Adenubi O.T., Morar-Leather D., Fasina F.O. (2017). Bacterial pathogens of pigs with particular reference to Escherichia coli: A systematic review and meta-analysis. J. Vet. Med. Anim. Health.

[4] Sora V.M., Meroni G., Martino P.A., Soggiu A., Bonizzi L., Zecconi A. (2021). Extraintestinal pathogenic Escherichia coli: Virulence factors and antibiotic resistance. Pathogens.

[5] Liu F., Ma J., Sui L., Wang W., Xiao Y., Dong Q., Xiao X. (2025). Unraveling the microbial contamination characteristics of pork farms and disinfection efficacy assessment via high-throughput sequencing. Anim. Dis..

[6] Li X., Hu H. (2024). Population structure and antibiotic resistance of swine extraintestinal pathogenic Escherichia coli from China. Nat. Commun..

[7] Manges A.R., Geum H.M., Guo A., Edens T.J., Fibke C.D., Pitout J.D. (2019). Global extraintestinal pathogenic Escherichia coli (ExPEC) lineages. Clin. Microbiol. Rev..

[8] Hao L., Huang W., Guo Y., Liu X., Wu J., Zhu L., Pan C., Wang H. (2025). A bioconjugate vaccine against extra-intestinal pathogenic Escherichia coli (ExPEC). Vaccines.

[9] Jansen K.U., Knirsch C., Anderson A.S. (2018). The role of vaccines in preventing bacterial antimicrobial resistance. Nat. Med..

[10] Micoli F., Costantino P., Adamo R. (2018). Potential targets for next generation antimicrobial glycoconjugate vaccines. FEMS Microbiol. Rev..

[11] Huang X., Wang X., Zhang J., Xia N., Zhao Q. (2017). Escherichia coli-derived virus-like particles in vaccine development. NPJ Vaccines.

[12] Kowarik M., Wetter M., Haeuptle M.A., Braun M., Steffen M., Kemmler S., Ravenscroft N., De Benedetto G., Zuppiger M., Sirena D. (2021). The development and characterization of an E. coli O25B bioconjugate vaccine. Glycoconj. J..

[13] Qiu L., Chirman D., Clark J.R., Xing Y., Hernandez Santos H., Vaughan E.E., Maresso A.W. (2024). Vaccines against extraintestinal pathogenic Escherichia coli (ExPEC): Progress and challenges. Gut Microbes.

[14] Zhou G., Wang Q., Wang Y., Wen X., Peng H., Peng R., Shi Q., Xie X., Li L. (2023). Outer membrane porins contribute to antimicrobial resistance in gram-negative bacteria. Microorganisms.

[15] Lin J., Huang S., Zhang Q. (2002). Outer membrane proteins: Key players for bacterial adaptation in host niches. Microbes Infect..

[16] Masi M., Winterhalter M., Pagès J.-M. (2019). Outer membrane porins. Bacterial Cell Walls and Membranes.

[17] Li L., Xu X., Cheng P., Yu Z., Li M., Yu Z., Cheng W., Zhang W., Sun H., Song X. (2025). Klebsiella pneumoniae derived outer membrane vesicles mediated bacterial virulence, antibiotic resistance, host immune responses and clinical applications. Virulence.

[18] Elshobary M.E., Badawy N.K., Ashraf Y., Zatioun A.A., Masriya H.H., Ammar M.M., Mohamed N.A., Mourad S., Assy A.M. (2025). Combating antibiotic resistance: Mechanisms, multidrug-resistant pathogens, and novel therapeutic approaches: An updated review. Pharmaceuticals.

[19] Wang W., Zhou X., Sang Y., Liang X., Liu J., Pan S., Wang L. (2021). Identification of a specific surface epitope of OmpC for Escherichia coli O157: H7 with protein topology facilitated affinity mass spectrometry. Appl. Microbiol. Biot..

[20] Igomu E.E., Mamman P.H., Adamu J., Muhammad M., Woziri A.O., Sugun M.Y., Benshak J.A., Anyika K.C., Sam-Gyang R., Ehizibolo D.O. (2025). Immunoinformatics design of a novel multiepitope vaccine candidate against non-typhoidal salmonellosis caused by Salmonella Kentucky using outer membrane proteins A, C, and F. PLoS ONE.

[21] Kwang J., He F. (2013). Microbes and livestock. Microbial Biotechnology: Principles and Applications.

[22] Nikaido H., Wu H.C. (1984). Amino acid sequence homology among the major outer membrane proteins of Escherichia coli. Proc. Natl. Acad. Sci. USA.

[23] Wang X., Guan Q., Wang X., Teng D., Mao R.-Y., Yao J., Wang J. (2015). Paving the way to construct a new vaccine against Escherichia coli from its recombinant outer membrane protein C via a murine model. Process Biochem..

[24] Pande J., Szewczyk M.M., Grover A.K. (2010). Phage display: Concept, innovations, applications and future. Biotechnol. Adv..

[25] Rao V.B. (2025). Bacteriophage T4 genome packaging: Mechanism and application. EcoSal Plus.

[26] Gamkrelidze M., Dąbrowska K. (2014). T4 bacteriophage as a phage display platform. Arch. Microbiol..

[27] Dąbrowska K., Miernikiewicz P., Piotrowicz A., Hodyra K., Owczarek B., Lecion D., Kaźmierczak Z., Letarov A., Górski A. (2014). Immunogenicity studies of proteins forming the T4 phage head surface. J. Virol..

[28] Liu C., Chen Z., Tan C., Liu W., Xu Z., Zhou R., Chen H. (2012). Immunogenic characterization of outer membrane porins OmpC and OmpF of porcine extraintestinal pathogenic Escherichia coli. FEMS Microbiol. Lett..

[29] Tehrani S.S., Jahangiri A., Taheri-Anganeh M., Maghsoudi H., Khalili S., Fana S.E., Maniati M., Amani J. (2020). Designing an outer membrane protein (Omp-W) based vaccine for immunization against Vibrio and Salmonella: An in silico approach. Recent Pat. Biotechnol..

[30] Chen T., Wang C., Hu L., Lu H., Song F., Zhang A., Wang X. (2021). Evaluation of the immunoprotective effects of IF-2 GTPase and SSU05-1022 as a candidate for a Streptococcus suis subunit vaccine. Future Microbiol..

[31] Zhu J., Tao P., Mahalingam M., Rao V.B. (2020). Preparation of a Bacteriophage T4-based Prokaryotic-eukaryotic Hybrid Viral Vector for Delivery of Large Cargos of Genes and Proteins into Human Cells. Bio Protoc..

[32] Zhu J., Ananthaswamy N., Jain S., Batra H., Tang W.C., Rao V.B. (2022). CRISPR Engineering of Bacteriophage T4 to Design Vaccines Against SARS-CoV-2 and Emerging Pathogens. Methods Mol. Biol. (Clifton N.J.).

[33] Zhu J., Ananthaswamy N. (2021). A universal bacteriophage T4 nanoparticle platform to design multiplex SARS-CoV-2 vaccine candidates by CRISPR engineering. Sci. Adv..

[34] Dong J., Chen C., Liu Y., Zhu J., Li M., Rao V.B., Tao P. (2021). Engineering T4 Bacteriophage for In Vivo Display by Type V CRISPR-Cas Genome Editing. ACS Synth. Biol..

[35] Tao P., Li Q., Shivachandra S.B., Rao V.B. (2017). Bacteriophage T4 as a nanoparticle platform to display and deliver pathogen antigens: Construction of an effective anthrax vaccine. Recombinant Virus Vaccines: Methods and Protocols.

[36] Cao N., Li Y., Zhao Q., Yao M., Ren X., Tian L., Hu Z., Diao F., Li H., Lu Z. (2025). Self-Assembled Nanoparticle Vaccines Elicit Robust Protective Immune Responses against Type O Foot-and-Mouth Disease Virus Infection. Acs Nano.

[37] Prejit N., Agarwal R.K., Porteen K., Dubal Z.B., Asha K., Shweta S., Ripan B. (2013). Evaluation of recombinant outer membrane protein based vaccine against Salmonella Typhimurium in birds. Biol. J. Int. Assoc. Biol. Stand..

[38] Arockiasamy A., Krishnaswamy S. (2000). Homology model of surface antigen OmpC from Salmonella typhi and its functional implications. J. Biomol. Struct. Dyn..

[39] Hu D., Qian P., Gao D., Li X., Wang L., Ji H., Wang S., Li X. (2024). Characterization and genomics analysis of phage PGX1 against multidrug-resistant enterotoxigenic E. coli with in vivo and in vitro efficacy assessment. Anim. Dis..

[40] Liu S., Lin M., Zhou X. (2025). T4 Phage Displaying Dual Antigen Clusters Against H3N2 Influenza Virus Infection. Vaccines.

[41] Zhu J., Tao P., Chopra A.K., Rao V.B. (2024). Bacteriophage T4 as a Protein-Based, Adjuvant- and Needle-Free, Mucosal Pandemic Vaccine Design Platform. Annu. Rev. Virol..

[42] Pokharel P., Dhakal S., Dozois C.M. (2023). The Diversity of Escherichia coli Pathotypes and Vaccination Strategies against This Versatile Bacterial Pathogen. Microorganisms.

[43] Smith J.L., Fratamico P.M., Gunther N.W. (2007). Extraintestinal pathogenic Escherichia coli. Foodborne Pathog. Dis..

[44] Fajgenbaum David C., June Carl H. (2020). Cytokine Storm. N. Engl. J. Med..

[45] Plotkin S.A., Plotkin S.A. (2008). Correlates of Vaccine-Induced Immunity. Clin. Infect. Dis..

