# Supplementary material for: Immune Protection Effect of an OmpC-Recombinant T4 Bacteriophage Vaccine Against Infection Caused by Extraintestinal Pathogenic Escherichia coli in Mice
Source: Vaccines (Basel). 2026 Apr 24;14(5):383. doi: 10.3390/vaccines14050383 (PMC13211530; doi:10.3390/vaccines14050383)
Supplement: Supplementary file 1 [file vaccines-4243614-supplementary.pdf › vaccines-4243614-supplementary.pdf]

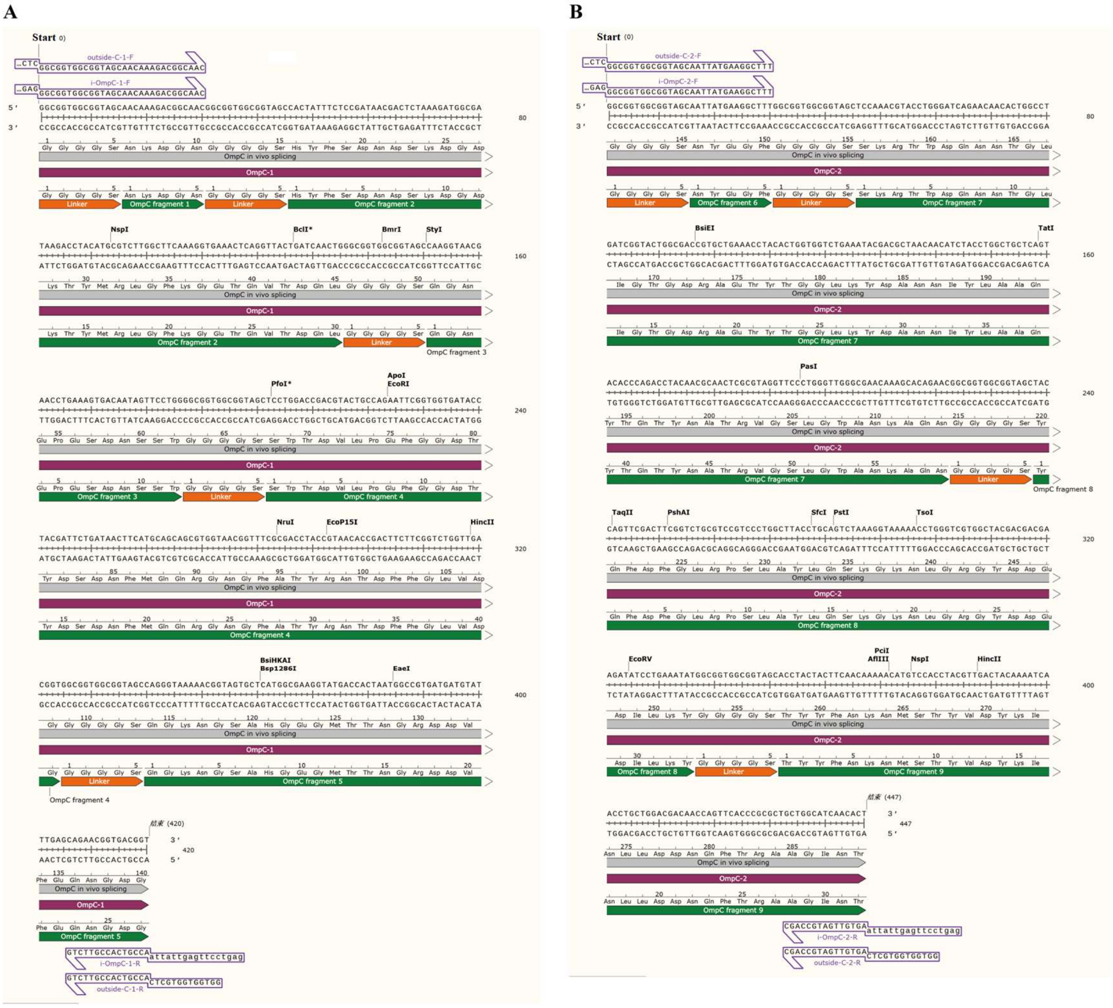

Figure S1. OmpC antigen epitope sequencing results. (A) OmpC-1;(B) OmpC-2;
